# Primary Cutaneous Lymphomas in Thailand: A 10-Year Retrospective Study

**DOI:** 10.1155/2021/4057661

**Published:** 2021-06-11

**Authors:** Chutipon Pruksaeakanan, Phurichaya Teyateeti, Poramin Patthamalai, Janista Thumrongtharadol, Manasmon Chairatchaneeboon

**Affiliations:** ^1^Department of Dermatology, Faculty of Medicine Siriraj Hospital, Mahidol University, Bangkok 10700, Thailand; ^2^Chulabhorn Hospital, HRH Princess Chulabhorn College of Medical Science, Chulabhorn Royal Academy, Bangkok 10210, Thailand

## Abstract

**Background:**

Primary cutaneous lymphomas (PCLs) refer to cutaneous lymphomas that primarily develop in the skin with no evidence of extracutaneous disease at the time of diagnosis. The epidemiological and clinical data of PCLs in Thailand are lacking.

**Objectives:**

To evaluate the frequency, demographic data, and clinical characteristics of different subtypes of PCLs in a tertiary care university hospital.

**Methods:**

In total, 137 patients with PCLs diagnosed in our hospital in 2008–2017 were retrospectively reviewed.

**Results:**

Of the 137 patients, 57 (41.6%) were male and 80 (58.4%) were female (M : F = 1 : 1.4). The median age at diagnosis was 40 years. Most patients (134, 97.8%) had cutaneous T-cell lymphomas (CTCLs). Three patients (2.2%) had cutaneous B-cell lymphomas (CBCLs). The most common subtype was mycosis fungoides (MF) (67.9%), followed by subcutaneous panniculitis-like T-cell lymphoma (SPTCL) (21.2%), primary cutaneous anaplastic large cell lymphoma (pcALCL) (3.6%), lymphomatoid papulosis (LyP) (1.5%), primary cutaneous gamma/delta T-cell lymphoma (pcGDTCL) (1.5%), Sézary syndrome (SS) (0.7%), extranodal NK/T-cell lymphoma, nasal type (ENKTCL-NT) (0.7%), primary cutaneous peripheral T-cell lymphoma, not otherwise specified (pcPTCL-NOS) (0.7%), primary cutaneous diffuse large B-cell lymphoma, leg type (pcDLBCL-LT) (1.5%), and primary cutaneous follicle center lymphoma (pcFCL) (0.7%). Most patients with MF presented with early-stage disease (84.0%), with hypopigmented MF the most common variant (42.6%).

**Conclusions:**

Compared to earlier Caucasian and Asian studies, the present study revealed a higher proportion of CTCL patients with a younger age at onset and a female predominance. MF was the most common CTCL subtype, followed by SPTCL. More than 80% of MF patients were diagnosed at an early stage.

## 1. Introduction

Primary cutaneous lymphomas (PCLs) are rare disorders, which refer to a group of extranodal non-Hodgkin lymphomas (NHLs) that primarily occur in the skin without evidence of extracutaneous disease at the time of diagnosis [1]. PCLs are the second most common extranodal NHLs after gastrointestinal lymphomas [2]. The estimated annual incidence of PCLs is approximately 1 : 100,000 [2, 3]. According to the World Health Organization-European Organization for Research and Treatment of Cancer (WHO-EORTC) classification for cutaneous lymphomas published in 2005 and updated in 2018, PCLs can be divided into 2 main categories: cutaneous T-cell lymphomas (CTCLs) and cutaneous B-cell lymphomas (CBCLs) [1, 4]. PCLs may resemble their nodal counterparts in clinical and immunohistopathological findings, but differ in terms of their clinical course, management, and prognosis. They tend to have a more indolent course and better prognosis for the most part. Therefore, the differentiation between primary and secondary cutaneous NHLs is extremely important.

CTCLs are the most common subtype of PCLs accounting for 75–80% of PCLs [1, 4]. Mycosis fungoides (MF) is the most common subtype, constituting 47–62% of CTCLs [4, 5]. Other subtypes of CTCLs vary among nationalities and geographical areas, such as subcutaneous panniculitis-like T cell lymphoma (SPTCL), which is quite rare among Caucasian populations [6, 7] but more common in Asian populations [8, 9].

CBCLs account for 20–25% of PCLs in Western countries. There are 3 main subtypes of primary cutaneous B-cell lymphomas: primary cutaneous follicle center lymphoma (pcFCL), primary cutaneous marginal zone lymphoma (pcMZL), and primary cutaneous diffuse large B-cell lymphoma, leg type (pcDLBCL-LT). The prevalence of CBCLs is relatively lower in Asian compared to Western populations [6, 8–10].

Currently, epidemiological and clinical data of PCLs in Southeast Asia remain sparse and indeed have not been previously reported in Thailand. Therefore, we aimed to study the frequency and demographic and clinical characteristics of the different subtypes of PCLs in our center, a tertiary care university hospital in Thailand.

## 2. Methods

This retrospective study was conducted at the Faculty of Medicine, Siriraj Hospital, Mahidol University. Patients diagnosed with cutaneous lymphomas from January 2008 to December 2017 were included. Patients with a history of extracutaneous lymphoma at the time of diagnosis or an incomplete history in the medical records were excluded. The diagnosis of PCLs was confirmed clinically and immunohistopathologically according to the WHO-EORTC classification for cutaneous lymphomas [1, 4]. Clinical stages and TNMB classification of patients with MF and Sézary syndrome (SS) were identified using the International Society for Cutaneous Lymphomas (ISCL) and the EORTC proposal in 2007, which was modified in 2011 [11, 12].

Data collection was performed by a review of the hospital electronic medical records. Demographic data and clinical characteristics, including age at diagnosis, sex, morphology, anatomical site of the lesions, TNMB classification and stage, and nodal and extracutaneous involvement, were recorded. This study was approved by the Siriraj Institutional Review Board ethics committee (Si 229/2019).

## 3. Results

We identified a total of 137 patients diagnosed with PCLs from 2008 to 2017.  Table 1 summarizes the demographic data and clinical characteristics of the PCL patients in our study. There were 57 (41.6%) males and 80 (58.4%) females, exhibiting a female predominance (M : F = 1 : 1.4). The median age at disease onset was 35 years (range 1–80 years). The median age at diagnosis was 40 years (range 1–83 years). CTCLs were the most common group of PCLs, comprising 134 (97.8%) patients, whereas only 3 (2.2%) patients were diagnosed with CBCLs. MF was the most common CTCL subtype, accounting for 67.9% of PCLs. Interestingly, SPTCL was the second most common CTCL subtype (21.2%), followed by pcALCL (3.6%). Regarding CBCL patients, 2 patients had pcDLBCL-LT and another one had pcFCL.

**Figure 1:**
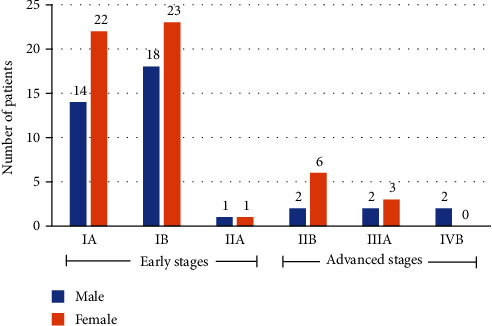
Clinical stages of 94 patients with mycosis fungoides and Sézary syndrome.

In MF/SS, the most common subtype of PCLs, there were 93 MF patients and 1 SS patient. Of these 94 patients, 39 (41.5%) were male and 55 (58.5%) were female (M : F = 1 : 1.4). The median age at disease onset was 34.5 years (range 1–80 years) and the median age at diagnosis was 40 years (range 1–83 years).  Figure 1 summarizes the clinical stages of the patients. In total, 79 (84.0%) patients were diagnosed with early-stage MF (stage IA–IIA).

The clinical characteristics by clinicopathological variants of patients with MF/SS are summarized in Table 2. The most common MF variant was hypopigmented MF (HMF), which accounted for 42.6% of cases. A female predominance was observed in classical, HMF, and folliculotropic MF (FMF), whereas poikilodermatous and erythrodermic MF occurred without gender predilection. The median age at the onset of symptoms and at diagnosis was younger in the hypopigmented (21.5, 28 years) and poikilodermatous variants (24, 39 years). All patients with HMF had only patch lesions. The most common sites of involvement were the trunk and lower extremities (79.8%), followed by the upper extremities (72.3%). Lesions on the head and neck were observed in 19.1% of cases, most frequently in patients with FMF subtype.

Among the 29 patients with SPTCL, the second most common subtype, 11 (37.9%) were male and 18 (62.1%) were female (M : F = 1 : 1.6). The median age of disease onset was 31 years (range 9–61 years). The median age at diagnosis was also 31 years (range 11–61 years). Most patients presented with nodules (79.3%), followed by plaques (24.1%) and patches (3.4%). The most common sites of involvement were the trunk and extremities (Table 3). In our study, the most common extracutaneous manifestations reported in SPTCL patients were B-symptoms (86.2%). Laboratory abnormalities included elevated LDH (82.8%), anemia (72.4%), leukopenia (65.5%), and elevated liver enzymes (55.2%). Four patients (13.8%) had hemophagocytic syndrome (HPS) or hemophagocytic lymphohistiocytosis (HLH).

## 4. Discussion

To date, only limited studies have reported on the epidemiology and clinical characteristics of PCLs in Southeast Asia. PCLs were recognized as the second most common extranodal NHLs after gastrointestinal lymphomas, according to most studies [2]. In 1998, researchers from Thailand reported that cutaneous lymphomas were the third most common extranodal NHLs, following upper aerodigestive tract and gastrointestinal tract [13]. However, epidemiological data on PCLs are rare and have not previously been investigated in Thailand. To the best of our knowledge, this is the first study focused on the frequency, demographic data and clinical characteristics of PCLs in Thailand. Our hospital is one of the largest tertiary care centers that accepts patient referrals from all regions of Thailand. Our study might represent the epidemiology of PCLs in Thailand. We also compared our results to previous national data from several countries and regions (Table 4).

Of the 137 PCL patients, we found that the frequency of CTCLs (97.8%) was higher than in most previous studies reported worldwide [3–6, 8–10, 14–16]. This finding is consistent with those reported in studies from China (94.5%) [10], Iran (96%) [17], Argentina (93%) [16], and Taiwan (92.3%) [18], but relatively higher than those from Japan (85.7%) [8] and Korea (88%) [9]. Our study emphasizes that non-Caucasian populations appeared to have a higher proportion of CTCLs than those of Caucasian populations (USA, 72.4% [6]; Netherlands and Austria, 78% [4]; Germany, 85% [5]; Switzerland, 72% [15]; Italy, 78.7% [3]). In contrast, the incidence of CBCLs (2.2%) was much lower in our study than that reported in studies from Western countries. The median age at diagnosis (40 years) of patients with PCLs overall in our study was younger than those reported in many previous studies, particularly from Western countries such as Switzerland (56 years) [15] and Italy (64 years) [3], but was close to those reported in studies from China (44.5 years) [10] and Iran (36 years) [17]. This could be explained by a high rate of HMF, a variant particularly affects young adults, in this study. However, the median age of CBCL patients (72 years) was considerably higher, compared with CTCL patients (39 years). Interestingly, we noticed a female predominance (M : F = 1 : 1.4) in almost all PCL subtypes, except for pcALCL. This finding was different from most earlier studies that reported a male predominance, but similar to the studies of a female predominance from Switzerland (M : F = 1 : 1.4) [15] and Iran (M : F = 1 : 1.2) [17].

MF/SS was the most common subtype of CTCLs observed in our study. The median age at onset (34.5 years) and at diagnosis (40 years) were younger than those reported in Western countries (57–61 years) [19–21]. Wilson et al. postulated that the age at presentation of patients with MF in non-white racial populations was younger than in whites [22]. We also found that HMF was the most common MF variant with a youngest median age at diagnosis (28 years). Our findings concurred with previous studies from Singapore, which demonstrated a young age of patients at the diagnosis of MF/SS and a high frequency of HMF. However, the median age of patients with HMF in Singapore was younger than in our study, according to reports by Tan et al. (17 years) [23] and Lim et al. (26 years) [24]. Our study supports the suggestion from previous data that HMF is predilected for dark-skinned and Asian patients, and it most commonly affects young adults and children [25]. All HMF patients in this study presented with patch lesions, which correlated with their excellent prognosis.

In terms of clinical staging, most MF/SS patients (84%) in the present study were diagnosed at early-stage, similar to previous studies from several countries, for instance, Taiwan (82.6%) [18], Iran (86.4%) [17], and Italy (87.2%) [3]. Early disease detection may be attributed to the development of advanced laboratory techniques, including genetics and molecular biology, and the rise in awareness among dermatologists and pathologists about PCLs in the recent years.

SPTCL is a rare subtype of CTCLs that preferentially involves subcutaneous tissue. We demonstrated that it predominantly affected young adult females with a median age at diagnosis of 31 years, similar to a previous study [26]. SPTCL constitutes approximately 1% of PCLs in Europe, but it is more common in Asia [8, 9]. According to Park et al., the frequency of SPTCL in Korea was 10.4% of PCLs [27]. Interestingly, SPTCL was the second most common subtype (21.2%) of PCLs demonstrated in our study, which is a higher figure than we found in other studies from the literature review. An earlier study on mature T-cell and NK-cell lymphomas conducted in our hospital reported that the prevalence of SPTCL was higher than in other studies as well [28]. B-symptoms were observed in 86.2% of SPTCL patients in the present study, which was a similar rate to those reported in Thailand (87.5%) by Rutnin et al. [29] and in Japan (81%) by Ohtsuka et al. [30]. However, the prevalence of B-symptoms in our study was higher than in Europe (59%) [26]. We observed that HPS developed in 13.8% of SPTCL cases. Studies by Willemze et al. [26], Ohtsuka et al. [30], Rutnin et al. [29], and Lee et al. [31] exhibited wide variations of the HPS associated with SPTCL in Europe (17%), Japan (45%), Thailand (37.5%), and Korea (14%), respectively. In addition, laboratory abnormalities, including elevated LDH, elevated liver enzymes, anemia, and leukopenia, were observed, similar to in previous reports [26, 29, 31, 32].

The occurrence of primary cutaneous CD30^+^ lymphoproliferative disorders (pcCD30^+^ LPDs) varied from 3% to 22% in earlier studies from different geographical areas (Table 4). Among Asian countries, Korea and Taiwan reported a high rate of pcCD30+ LPDs at about 20% of PCLs, but we found this condition only in 5.1% of PCLs. According to our study, the prevalence of pcALCL was 3.6% which was similar to the reports from China and Iran, but it was lower than several reports from Western and Asian countries. Notably, compared with other countries, Korea had considerably higher rate (11.1%) of pcALCL. The proportion of patients with pcALCL in our study was higher than LyP, similar to in studies from Japan and Korea. LyP is a self-limiting condition that can mimic many inflammatory dermatoses and other lymphomas, both clinically and histopathologically. In addition, more histologic subtypes of LyP have been established over time, even up to recently [1]. As a result, LyP may be underdiagnosed in some circumstances, such as the lack of experienced dermatopathologists or cutaneous lymphoma experts in the area.

The prevalence of other rare subtypes of PCLs can be significantly different among centers. Regarding extranodal NK/T-cell lymphoma, nasal type (ENKTCL-NT), several authors have indicated a higher frequency of this condition in Asian countries compared to in Western countries and a strong association between Epstein-Barr virus (EBV) and ENKTCL in Asian populations [33–35]. However, we found only 1 case (0.7%) of ENKTCL-NT in our study, which was lower than reported in other Asian countries [8–10, 36]. In our center experience, many patients with ENKTCL-NT present with extracutaneous manifestations, this can be the explanation of a low rate of primary cutaneous ENKTCL-NT. After a thorough review, we also observed differences in the proportions of ENKTCL-NT, even among studies from the same country. For example, in 2 previous studies from Korea, the incidence of ENKTCL-NT was 16.7% of PCL patients in a study by Park et al. [27] yet 5.4% in another study by Lee et al. [9]. From this point of view, we believe that multiple factors play a role in the pathogenesis of various subtypes of PCLs, besides genetics, ethnicity, and geographical areas.

Notably, Japan has a high prevalence of adult T-cell leukemia/lymphoma (ATLL) compared with other countries or regions, including Asian countries, because it is an endemic area of human T-cell lymphotropic virus type 1 (HTLV-1), the virus that causes ATLL [8, 37]. In contrast, the prevalence of HTLV-1 infection in Thailand is extremely low, which could explain the absence of patients with ATLL in this study [38].

In our study, CBCLs were much rarer (2.2%), and the pattern of CBCL subtypes was somewhat different, compared to previous studies. pcDLBCL-LT was the most common subtype of CBCLs in the present study, similar to that found in some earlier studies in Asia and the United States; whereas in Europe, pcFCL and pcMZL are more common (Table 4). Several authors discovered an association between pcMZL in Europe and *Borrelia burgdorferi* infection or Lyme disease [39, 40]. Thailand is not an endemic area of Lyme disease, and there are no tick species that are *Borrelia* vectors found in this region [41]. Besides, pathologists occasionally experience challenges to distinguish pcMZL from cutaneous lymphoid hyperplasia (B-cell pseudolymphoma), despite the available immunohistochemical markers [42]. These could be the explanations for the low frequency of CBCLs and the absence of pcMZL in the present study.

The limitations of our study were a single-center retrospective study design and the lack of review in histopathology, although we excluded cases in which pathologists at our hospital did not confirm the diagnosis.

## 5. Conclusions

In conclusion, this is the first study to evaluate the frequency, demographic data, and clinical characteristics of different subtypes of PCLs in Thailand. We reported a high ratio of CTCLs, especially MF and SPTCL, with a female predominance and younger age of patients. The majority of MF patients had early-stage disease, and HMF was the most common subtype. Since PCLs are rare in the Thai population, a national PCLs registry in Thailand is our future goal.

## Figures and Tables

**Table 1:** Frequency of primary cutaneous lymphomas by sex, median age at onset, and at diagnosis of the diseases.

	Total *N* (%)	Male *N*	Female *N*	M : F ratio	Age at onset, median (range), years	Age at diagnosis, median (range), years
Total	137 (100)	57	80	1 : 1.4	35 (1-80)	40 (1-83)
Cutaneous T-cell and NK-cell lymphomas	134 (97.8)	56	78	1 : 1.4	35 (1-80)	39 (1-83)
Mycosis fungoides	93 (67.9)	38	55	1 : 1.4	34 (1-80)	40 (1-83)
Sézary syndrome	1 (0.7)	1	0	-	64 (64)	66 (66)
Primary cutaneous anaplastic large cell lymphoma	5 (3.6)	3	2	1.5 : 1	51 (11-66)	51 (11-66)
Lymphomatoid papulosis	2 (1.5)	1	1	1 : 1	52.5 (42-63)	52.5 (42-63)
Subcutaneous panniculitis-like T-cell lymphoma	29 (21.2)	11	18	1 : 1.6	31 (9-61)	31 (11-61)
Extranodal NK/T-cell lymphoma, nasal type	1 (0.7)	1	0	-	54 (54)	57 (57)
Primary cutaneous gamma/delta T-cell lymphoma	2 (1.5)	0	2	-	41.5 (13-70)	42.5 (13-72)
Primary cutaneous peripheral T-cell lymphoma, not otherwise specified	1 (0.7)	1	0	-	58 (58)	60 (60)
Cutaneous B-cell lymphomas	3 (2.2)	1	2	1 : 2	72 (67-74)	72 (68-74)
Primary cutaneous diffuse large B-cell lymphoma, leg type	2 (1.5)	1	1	1 : 1	69.5 (67-72)	70 (68-72)
Primary cutaneous follicle center lymphoma	1 (0.7)	0	1	-	74 (74)	74 (74)

**Table 2:** Clinical characteristics of different variants of mycosis fungoides **(**MF**)** and Sézary syndrome **(**SS**).**

	Classical MF	Hypopigmented MF	Poikilodermatous MF	Folliculotropic MF	Erythrodermic MF	Sézary syndrome	All MF/SS
*N* (%)	36 (38.3)	40 (42.6)	8 (8.5)	5 (5.3)	4 (4.3)	1 (1.1)	94 (100)
Sex							
Male	15	15	4	2	2	1	39
Female	21	25	4	3	2	0	55
Male : female	1 : 1.4	1 : 1.7	1 : 1	1 : 1.5	1 : 1	Male	1 : 1.4
Age (years)							
Onset	49	21.5	24	62	63	64	34.5
Diagnosis	49.5	28	39	62	67	66	40
Morphology, *N* (%)							
Patch	25 (69.4)	40 (100)	7 (87.5)	3 (60.0)	2 (50.0)	1 (100)	78 (83.0)
Plaque	23 (63.9)	0 (0)	4 (50.0)	5 (100)	4 (100)	1 (100)	37 (39.4)
Tumor	6 (16.7)	0 (0)	0 (0)	1 (20.0)	2 (50.0)	0 (0)	9 (9.6)
Ulcer	3 (8.3)	0 (0)	0 (0)	0 (0)	0 (0)	0 (0)	3 (3.2)
Erythroderma	0 (0)	0 (0)	0 (0)	1 (20.0)	4 (100)	1 (100)	6 (6.4)
Location, *N* (%)							
Head and neck	6 (16.7)	3 (7.5)	1 (12.5)	4 (80.0)	3 (75.0)	1 (100)	18 (19.1)
Trunk	30 (83.3)	31 (77.5)	8 (100)	1 (20.0)	4 (100)	1 (100)	75 (79.8)
Upper extremities	29 (80.6)	30 (75.0)	4 (50.0)	1 (20.0)	4 (100)	1 (100)	68 (72.3)
Lower extremities	29 (80.6)	34 (85.0)	5 (62.5)	2 (40.0)	4 (100)	1 (100)	75 (79.8)

**Table 3:** Morphologies and anatomical locations of primary cutaneous T-cell and B-cell lymphomas other than mycosis fungoides and Sézary syndrome.

	Cutaneous T-cell and NK-cell lymphomas						Cutaneous B-cell lymphomas	
	pcALCL (*N* = 5)	LyP (*N* = 2)	SPTCL (*N* = 29)	ENKTCL-NT (*N* = 1)	pcGDTCL (*N* = 2)	pcPTCL-NOS (*N* = 1)	pcDLBCL-LT (*N* = 2)	pcFCL (*N* = 1)
Morphology, *N* (%)								
Patch	0 (0)	0 (0)	1 (3.4)	0 (0)	0 (0)	0 (0)	0 (0)	0 (0)
Plaque	0 (0)	0 (0)	7 (24.1)	0 (0)	1 (50.0)	0 (0)	1 (50.0)	1 (100)
Papule	0 (0)	2 (100)	0 (0)	0 (0)	0 (0)	1 (100)	0 (0)	0 (0)
Nodule	4 (80.0)	1 (50.0)	23 (79.3)	1 (100)	2 (100)	1 (100)	2 (100)	0 (0)
Ulcer	1 (20.0)	2 (100)	0 (0)	1 (100)	1 (50.0)	0 (0)	0 (0)	0 (0)
Location, *N* (%)								
Head and neck	2 (40.0)	1 (50.0)	1 (3.4)	0 (0)	1 (50.0)	1 (100)	0 (0)	1 (100)
Trunk	4 (80.0)	2 (100)	22 (75.9)	0 (0)	2 (100)	1 (100)	1 (50.0)	0 (0)
Upper extremities	0 (0)	2 (100)	17 (58.6)	1 (100)	1 (50.0)	1 (100)	0 (0)	0 (0)
Lower extremities	2 (40.0)	1 (50.0)	25 (86.2)	0 (0)	2 (100)	1 (100)	1 (50.0)	0 (0)

pcALCL: primary cutaneous anaplastic large cell lymphoma; LyP: lymphomatoid papulosis; SPTCL: subcutaneous panniculitis-like T-cell lymphoma; ENKTCL-NT: extranodal NK/T-cell lymphoma, nasal type; pcGDTCL: primary cutaneous gamma/delta T-cell lymphoma; pcPTCL-NOS: primary cutaneous peripheral T-cell lymphoma, not otherwise specified; pcDLBCL-LT: primary cutaneous diffuse large B-cell lymphoma, leg type; pcFCL: primary cutaneous follicle center lymphoma.

**Table 4:** Comparison of the frequencies of primary cutaneous lymphomas **(**PCLs**)** in different countries around the world.

	Asians						Caucasians				
	Our study	Japan	Korea (PCLs only)	China	Taiwan	Iran	USA (PCLs only)	Netherland + Austria	Germany	Switzerland	Italy
Author, Year	Pruksaeakanan et al., 2021	Hamada et al., 2014, [8]	Lee et al., 2016, [9]	Shi et al., 2019, [10]	Lee et al., 2018, [18]	Naeini et al., 2015, [17]	Bradford et al., 2009, [6]	Willemze et al., 2005, [4]	Assaf et al., 2007, [5]	Jenni et al., 2011, [15]	Maurelli et al., 2018, [3]
Study period	2008-2017	2007-2011	2009-2013	2010-2018	2001-2010	2003-2013	2001-2005	1986-2002	1999-2004	1990-2009	2005-2015
Number of cases	137	1,733	333	850	91	99	3,827	1,905	998	263	141
Median age at diagnosis (yr)	40	65	49	44.5	NA	36	NA	NA	NA	56	64
Male : female	1 : 1.4	1.3 : 1	1.6 : 1	Male predominance	1.6 : 1	1 : 1.2	1.4 : 1	NA	NA	1 : 1.4	1.8 : 1
CTCLs	97.8%	85.7%	88.0%	94.8%	92.3%	96.0%	72.4%	77.5%	85.0%	72.0%	78.7%
Median age at diagnosis (yr)	39	64	47	44	50	36	NA	NA	NA	57.5	NA
Mycosis fungoides	67.9%	43.3%	49.0%	65.4%	57.1%	86.9%	38.9%	48.0%	62.0%	43.0%	50.3%
Sézary syndrome	0.7%	1.9%	0%	NA	2.2%	4.0%	0.9%	3.0%	2.0%	11.0%	2.8%
ATLL	0%	16.7%	0%	NA	0%	NA	0.1%	NA	NA	NA	NA
pcCD30^+^ LPDs	5.1%	12.0%	20.7%	11.0%	22.0%	3.0%	10.3%	20.0%	14.0%	13.0%	20.6%
pcALCL	3.6%	7.8%	11.1%	3.0%	7.7%	2.0%	NA	8.0%	7.0%	8.0%	5.7%
LyP	1.5%	3.8%	9.6%	8.0%	14.3%	1.0%	NA	12.0%	7.0%	5.0%	14.9%
SPTCL	21.6%	2.0%	4.5%	2.0%	2.2%	NA	0.6%	1.0%	NA	NA	0.7%
ENKTCL-NT	0.7%	2.0%	5.4%	5.0%	4.4%	1.0%	0.3%	<1%	NA	<1%	0.7%
pcGDTCL	1.5%	0.3%	1.8%	NA	0%	NA	NA	<1%	NA	NA	NA
pcAECD8^+^ cytotoxic TCL	0%	0.3%	0.6%	NA	0%	NA	NA	<1%	NA	NA	NA
pcCD4^+^ small/medium T-cell LPD	0%	1.4%	2.7%	NA	2.2%	NA	NA	2.0%	5.0%	3.0%	2.8%
pcPTCL-NOS	0.7%	5.8%	7.8%	NA	1.1%	1.0%	21.1%	2.0%	NA	2.0%	NA
CBCLs	2.2%	12.9%	11.7%	5.0%	7.7%	4.0%	27.4%	22.5%	15.0%	28.0%	21.3%
Median age at diagnosis (yr)	72	70	56	58	78	40.5	NA	NA	NA	53	65
pcMZL	0%	4.2%	4.5%	2.0%	3.3%	1.0%	7.2%	7.0%	3.9%	14%	2.8%
pcFCL	0.7%	2.1%	0.9%	0.7%	0%	2.0%	8.6%	11.0%	6.8%	8.0%	14.2%
pcDLBCL	1.5%	5.5%	6.3%	3.0%	4.4%	NA	11.6%	4.0%	1.7%	4.0%	4.3%

CTCL: cutaneous T-cell lymphoma; pcCD30^+^ LPDs: primary cutaneous CD30^+^ lymphoproliferative disorders; pcALCL: primary cutaneous anaplastic large cell lymphoma; LyP: lymphomatoid papulosis; SPTCL: subcutaneous panniculitis-like T-cell lymphoma; ENKTCL-NT: extranodal NK/T-cell lymphoma, nasal type; pcGDTCL: primary cutaneous gamma/delta T-cell lymphoma; pcAECD8^+^ cytotoxic TCL: primary cutaneous aggressive epidermotropic CD8^+^ cytotoxic T-cell lymphoma; pcCD4^+^ small/medium T-cell LPD: primary cutaneous CD4^+^ small/medium T-cell lymphoproliferative disorder; pcPTCL-NOS: primary cutaneous peripheral T-cell lymphoma, not otherwise specified; CBCL: cutaneous B-cell lymphoma; pcMZL: primary cutaneous marginal zone lymphoma; pcFCL: primary cutaneous follicle center lymphoma; pcDLBCL: primary cutaneous diffuse large B-cell lymphoma; NA: not available.

## Data Availability

The data used to support the findings of this study included within the article.
